# Atlanta Academy of Medicine

**Published:** 1871-10

**Authors:** Edwin P. Ingraham

**Affiliations:** Reporter


### ATLANTA ACADEMY OF MEDICINE.

EDWIN P. INGRAHAM, M.D., REPORTER.

August 11th, 1871.

Dr. Logan, President, in the chair.

Dr. Judson reported a case of "apparent death" of a new-born child. He used cold water, camphor, artificial respiration, without producing any effect. He then resorted to the warm bath, which soon resuscitated the child. Sixteen minutes elapsed from the birth of the child until it first respired. Dr. Judson reported the case for the purpose of calling attention to the efficacy of the warm bath in such cases. His attention had been directed to it, when a student, by an educated midwife, and he had, in a number of instances, had occasion to resort to its use, with favorable results, when other means failed.

Dr. Cumming remarked, that Dr. Judson's case recalled to his mind one which Dr. John Ross, of Toronto, Canada, and himself, treated several years ago. They continued to work with the child for fourteen hours after birth, before fully resuscitated.

Dr. Wilson.—Did fourteen hours elapse after birth before the child breathed?

Dr. Cumming.—It breathed feebly occasionally, and seemed to be dying.

Dr. Love stated, that Dr. E. T. McDaniel, of Camden, Alabama, in a paper read before the American Medical Association, (Transactions American Medical Association, vol. xx., 1869), recommends what he terms the warm cerebro-spinal bath, with artificial respiration, in congenital apnœa, and states that "it is not at all unusual to see, after a faithful perseverance with the method,—warm bath and artificial respiration,—the first independent respiratory movement take place twenty or thirty minutes after birth, and to have the case afterward do well. I remember one case in which the first inspiration took place thirty minutes after birth; another, ninety minutes; another, two and a half hours; and another, four hours."

Dr. C. Hanfield Jones had, about the same time, called the attention of the profession in Europe to the same or a similar plan of treatment. Dr. Love states that he has found this plan of treatment more satisfactory than any other he has been able to adopt. One case he reports, where the bath and artificial respiration had been kept up for *one hour and twenty minutes* before an independent respiration was established. This case occurred in 1859 or 1860. Since and before that time, he has relied with implicit confidence on this plan. He believes Dr. McDaniel to have been the first to call the attention of the profession to the subject in a regular paper.

Dr. Wilson has frequently used the warm bath, alternated with cold. In one case, he was fully an hour in resuscitating a child. He is satisfied that we should never give up without first trying the use of the warm, alternated with the cold bath.

Dr. Vance suggested that it was impossible for the heart's action to be suspended longer than two and a half minutes without death occuring. As long as the action of the heart could be detected, we should continue our exertions to resuscitate; but when the heart ceased to act, he deemed life extinct. The alternate use of the warm and cold bath, he believed, had been long recommended by teachers of obstetrics.

Dr. Cumming stated that lambs, in cold climates, frequently dropped in the cold, and when found apparently dead, were often restored by placing them before the fire—first the chest, and then back. He thought this same principle applicable in resuscitating children.

Dr. Logan exhibited some cloth tents made and presented to the Academy by Dr. V. H. Taliaferro, of Columbus, Ga. Among the advantages Dr. Taliaferro claims for the cloth tent over the sponge, for general use, are the following:

"1. Its cheapness, simplicity, and easy preparation. Any one, with a little practice, can make them. The strips of cloth should be from one-half to one inch wide, and rolled between the thumb and fore-finger. To make them sufficiently firm, they require to be rolled very tightly.

"2. The easy and thorough manner in which medication may be made to the cavity of the uterus and cervical canal—

separately, or to both at the same time. The point of the tent may be alone medicated, if it is desirable, or that portion only coming in contact with the cervix.

"3. It does not produce irritation and pain, and hence, is free from the serious and sometimes fatal inflammatory results of the sponge tent. It is soft, flexible, innoxious, and readily adapts itself to the shape of the uterine cavity.

"4. The large amount of medicament that may be thus applied to act so gradually on the uterine surface as to create no disturbances, such as uterine colics, inflammations, etc., that so frequently occur with the sponge.

"5. Its easy removal from the uterus, which the patient may do herself, if desirable, by means of the thread attached to the distal extremity. The removal of the sponge is often difficult and painful."

There are many other advantages possessed by this tent, which, by its daily use, will become apparent. The serous flow, by exosmosis, induced by this tent is not inferior to that of the sponge. It is far superior to the sponge when saturated with glycerine, or glycerine and carbolic acid. Dr. Taliaferro states that he is in the habit of using most frequently, as a medication with this tent: tincture of iodine, solution of iodine and bromine, carbolic acid, iodoform and glycerine, and occasionally had cauterized the uterine cavity with chromic acid, nitrate of silver, and carbolic acid, with this tent. When the chromic acid is used, it is necessary to first coat the tent with suet and then with the solution of the acid, as without this the tent will be slowly dissolved by the acid. When caustics are used, the tent is, of course, permitted to remain in contact with the uterine surface but a few moments. The cavity of the uterus should always be cleansed before using the caustics, else they are neutralized by the secretions before coming in contact with the mucous surface. He would take the liberty of advising great caution in the use of chromic acid to the *intra-uterine surface*, as in several instances he had witnessed its application followed by excessive and alarming nausea, vomiting and prostration, and this without pain. This condition has always resulted when the tents had not been used several times previous to the use of the acid. His opinion is, that previous to the application of any caustic to the uterine cavity, there should be produced a degree of *tolerance* upon the part of the uterus, by the use of the tents, saturated with glycerine, for several days in succession — the tents being renewed once in twenty-four hours. He had observed, when this had been done, no unpleasant results followed the use of the caustics Another advantage in preceding the caustic application by the tents saturated with glycerine is, that the cavity of the uterus may be always thoroughly washed out with a little warm water; he invariably does this, and has never had an untoward symptom. It is his custom even to *wash out* the uterus before *renewing* the medicated tent. The contact of a tent with the internal os for twenty-four hours so completely paralyzes its muscular fibres as to thoroughly disable its contractile powers for the time being. So long as this is the case, there is no danger from the injection of any amount of water or of medicated fluids (of proper strength) into the uterine cavity.

Dr. Logan stated that he had written to Dr. Taliaferro in reference to the *dilating* properties of the *cloth tent*, as a substitute for the sponge — to which Dr. Taliaferro replied:

"For all purposes, save that of a *quick dilatation* for exploration of the uterine cavity, the *cloth tent* is designed to take the place of all others. When internal medication to the cavity of the uterus is desired by means of injections, ointments, the cotton-wrapped probe, or the solid nitrate of silver, the cloth tent is preferable. Almost any desirable extent of dilatation may be obtained by increasing, from day to day, the size of the tent. When there is great irritability of the uterus, as is often the case, (making the sponge a remedy of danger), this tent is not only innoxious, but, saturated with glycerine, is a therapeutic agent of great power, inducing, by exosmosis, a free and constant flow of serum. Thus prepared, this tent may be used, from day to day, for ten or twelve days, or even longer. For purposes of internal medication, by injections or otherwise, the dilatation with this tent is quite sufficient. I often inject the cavity of the uterus after the use of a single tent for twenty-four hours. If, however, I find it necessary to begin with a very small tent, I use several, increasing the size and length in each one, before resorting to injections. In the use of injections in the uterine cavity, the extent of dilatation is not so important as that the contractile power of the internal os shall be *thoroughly paralyzed*, and that the nozzle of the syringe used shall not completely fill the cavity.

"Following the use of these tents, I *wash out* the uterine cavity as I would any other cavity, not being governed by the amount of water used, but continuing it until the cavity is thoroughly cleansed. I sometimes add a little salt to the water—sometimes, a little carbolic acid.

"As a therapeutic agent—a means of medicating the uterine cavity—this tent claims *superior* advantages over the medicated sponge, or the sea tangle, or the cotton-wrapped probe of Thomas, the glass rod of Simms, or the fenestrated tubes of Peasley.

"With the probes, or with Peasley's fenestrated tubes, the article used for medication is simply painted over the mucus surface, and, with either plan, the medication must be very uncertain and imperfect. With the cloth tent, we not only keep up a sufficient dilatation of the internal os, to insure a free exit for all discharges occasioned by the treatment, but we retain the remedy in contact with the cavity as long as we may desire, thereby inducing the requisite therapeutic action by diffusion of our remedy, as in the case of iodine, for example—slowly, gradually and thoroughly, and not only upon the mucous surface alone, but upon all the tissues of the uterus, by absorption. As before remarked, a very important advantage which this tent claims over all other plans of medicating the cavity of the uterus is, that it insures an *open canal*, thereby insuring a free exit for all discharges, and enabling us, upon each removal of the tent, to thoroughly cleanse the uterine cavity. Dr. Nott says, and truly: 'To my mind, one of the most important principles in the local treatment of endometritis, and one which I have before insisted on, is the cleansing of the organ of its foul secretions, by frequent ablutions with a syringe.'

"By the plan of Dr. Peasley with his fenestrated tubes, dilatation with the sponge, or his steel dilators, must precede his treatment, and so much irritation is consequently produced as to necessarily require long intervals between applications. Dr. Thomas, who seems to be fond of Peasley's steel dilators, says of a patient of his, upon whom he used three of these dilators *on Monday*: 'Tuesday evening, I was summoned to Staten Island, where I found the lady with violent pelvic peritonitis, of which she died on *Sunday* evening.' We not unfrequently have similar results by dilatation with the sponge. With the *cloth tent*, our treatment may be continuous for days in succession, thereby keeping up the impression our agents are designed to produce, and at the same time keeping up the *needful and absolutely necessary dilatation.* My own mind is satisfied that the bad results so often resulting from intra-uterine medication, is owing mainly to the application of remedies while the *internal os* is possessed of all its contractility. This power needs to be paralyzed, to insure the cleansing of the cavity, and the thorough and safe applications of remedial agents. The rapid and forcible dilatation by the sponge, or with Peasley's dilatators, is attended with *great danger*, and hence, as a preparatory step to intra-medication, will always be used with 'fear and trembling.' The *cloth tent*, saturated as we have directed, with glycerine, may be used with the most irritable uterus 'ad libitum,' and free from danger, and with a rapid relief of congestion and irritation."

